# Cacao Pod Husk Extract Phenolic Nanopowder-Impregnated Cellulose Acetate Matrix for Biofouling Control in Membranes

**DOI:** 10.3390/membranes11100748

**Published:** 2021-09-29

**Authors:** Yusuf Wibisono, Eka Mustika Diniardi, Dikianur Alvianto, Bambang Dwi Argo, Mochamad Bagus Hermanto, Shinta Rosalia Dewi, Nimatul Izza, Angky Wahyu Putranto, Saiful Saiful

**Affiliations:** 1 Department of Bioprocess Engineering, Brawijaya University, Jl. Veteran, Malang 65145, Indonesia; dwiargo@ub.ac.id (B.D.A.); shintarosalia@ub.ac.id (S.R.D.); izza_nimatul@ub.ac.id (N.I.); angkywahyu@ub.ac.id (A.W.P.); 2 MILI Institute for Water Research, Kawasan Industri Jababeka, Bekasi 17530, Indonesia; 3 Department of Agricultural and Biosystem Engineering, Brawijaya University, Jl. Veteran, Malang 65145, Indonesia; eka_ardi@yahoo.com (E.M.D.); dikianur@gmail.com (D.A.); mbhermanto@ub.ac.id (M.B.H.); 4 Department of Chemistry, Faculty of Mathematics and Natural Sciences, Syiah Kuala University, Banda Aceh 23111, Indonesia; saiful@unsyiah.ac.id

**Keywords:** composite membranes, cacao pod husk extract, cellulose acetate, phenolic nanoparticles, biofouling

## Abstract

The ultrafiltration membrane process is widely used for fruit juice clarification, yet the occurring of fouling promotes a decline in process efficiency. To reduce the fouling potential in the membrane application in food processing, the use of natural phenolic compounds extracted from cocoa pod husk is investigated. The cocoa pod husk extract (CPHE) was prepared in phenolic nanoparticles form and added into the polymer solution at varying concentrations of 0.5 wt%, 0.75 wt%, and 1.0 wt%, respectively. The composite membrane was made of a cellulose acetate polymer using DMF (dimethylformamide) and DMAc (dimethylacetamide) solvents. The highest permeability of 2.34 L m^−2^ h^−1^ bar^−1^ was achieved by 1.0 wt% CPHE/CA prepared with the DMAc solvent. CPHE was found to reduce the amount of *Escherichia coli* attached to the membranes by 90.5% and 70.8% for membranes prepared with DMF and DMAc, respectively. It is concluded that CPHE can be used to control biofouling in the membrane for food applications.

## 1. Introduction

In the food industry, membrane technology has been widely applied, for instance, in the process of clarification and increasing the concentration of beverages made from agricultural products [1,2]. Food elements and constituents could be separated by membranes, especially in the range of microfiltration and ultrafiltration membrane pores [3]. Compared to conventional technology (such as evaporation), this technology requires less labor and has a higher level of efficiency with shorter processing times and lower operating temperatures. As a result, operational costs can be decreased with better product quality [4].

The main problem in the membrane-based technology processes is fouling, which is largely caused by biological components such as bacteria, fungi, and algae, or biofouling [5]. Biofouling is defined as the accumulation of microorganisms, i.e., biofilm growth, on the membrane surface, which causes operational problems [6]. Moreover, biofouling can increase concentration polarization, which stimulates inorganic scaling [7].

Cellulose acetate (CA)-based polymers are commonly used materials to form membranes for aqueous applications [8,9]. CA membranes have advantages such as providing a high flux, high hydrophilicity, biocompatibility, and affordability [10]. However, the major drawback of CA is that it is easily degraded by cellulose enzymes released by microorganisms living on its surface [11]. Controlling biofouling in CA membranes is therefore very important because biofouling on the CA membrane surface not only reduces operational performance but also causes membrane damage [12].

Alternatives for reducing fouling in membranes have been investigated, such as utilizing chemical compounds or toxic substances, which possess antibacterial properties [13,14,15,16]. A more reliable and safer additive is adding a natural phenolic compound, which also has the ability to control biofouling in membranes [17]. Phenolic substances can be extracted from nature, such as *Moringa oleifera* seeds [18], olive leaves [19], longevity spinach [20], fig fruit [21], papaya [22], pineapple peel [23], or garlic bulb [24]. As the substance can be extracted from many plants, the use of agricultural product leftovers or biowaste is more advantageous compared with extracting from edible plants. Among the plant wastes that contain phenolic compounds is cacao pod husk (CPH), widely found from cacao plantations. Along with a ton of dried cocoa beans produced, at least 10 t of CPH remained [25]. As a country such as Indonesia produces more than 600 t of dry cocoa beans a year [26], approximately 6 million ton of fresh CPH are produced as biowaste. CPH is normally only piled up in the plantation and not utilized.

CPH contains phenolic compounds such as phenolic acids (p-hydroxybenzoic acid, protocatechuic acid, methyl salicylate, and salicyclic acid), flavonols (e.g., kaempferol and rhamnetin), and flavones (e.g., linarin, acatecin, and luteolin) [27,28]. CPH extract (CPHE) can inhibit some bacterial growth, e.g., *Escherichia coli*, *Bacillus subtilis*, and *Staphylococcus aureus* [29]. CPHE was also able to inhibit the growth of *Fusarium oxysporum* fungal pathogens [30]. CPHE can be utilized as a natural food preservative [31,32].

The use of phenolic compounds in the form of CPHE has never been utilized as an antibacterial additive in membrane preparation. This study, therefore, investigated the use phenolic nanopowder from CPHE impregnated in cellulose acetate polymer matrix. The effect of different concentration of CPHE phenolic nanopowder on the properties of CA membranes is investigated, especially the influence of the antibiofouling properties of the membranes. A CPHE phenolic nanopowder-impregnated CA membrane is expected to promote natural antibiofouling agents for microfiltration membranes utilized for food processing.

## 2. Materials and Methods

### 2.1. Materials

CPH was purchased from local cacao farmers in Indonesia. Cacao ripe fruit was harvested from a cacao plantation located approximately 650 m above sea level and picked up during the rainy season. CPH was derived from cacao ripe fruit that was harvested for no more than 3 days prior to the extraction process, to reduce seasonal and batch-to-batch variation [33] Raw CPH was washed using clean water and then chopped into 2 mm thickness. The CPH pellets were then dried in an oven at temperature of 50 °C for 24 h. Dried CPH pellet were then pulverized and sieved with a 100 mesh sieve to produce the CPH powder and then extracted. Cellulose acetate (CA) powder with 39.3–40.3 wt% acetyl content and average molecular weight (Mn) 30,000 as measured by gel permeation chromatography (GPC), dimethylformamide (DMF), and dimethylacetamide (DMAc) for membrane synthesis, and were supplied by Sigma-Aldrich (Merck, Darmstadt, Germany).

### 2.2. Preparation of CPHE

The extraction of CPHE was carried out by using the microwave-assisted extraction method, using 96% ethanol as the solvent, as reported previously [34,35]. The CPH powder weighed 25 grams and was added to 50 mL hexane. The solution was then mixed with a magnetic stirrer for 30 min until the solution was homogenous, and then allowed to stand for 15 min and decanted. This defatting process was repeated one more time. The material was then extracted using 100 mL ethanol in the microwave, set at power of 180 Watts (Samsung ME731K, Seoul, South Korea) for 4 min. 

Maceration continued for 24 h while occasionally being stirred. The material was then filtered using Whatman No. 1 filter paper and filtrate-1 was obtained. The residue from the filtering results was macerated by adding 50 mL of ethanol for 24 h and filtered to obtain filtrate-2. Both filtrate-1 and filtrate-2 were mixed and inserted into the rotary evaporator (IKA HB 10, Staufen, Germany) at a temperature of 40 °C at 65 rpm, until it became a thick solution. The concentrated extract was then dried by using a vacuum oven at a temperature of 50 °C and then mashed with a mortar. CPHE particles size was measured using a Zetasizer Nano (Malvern Panalytical Ltd, Malvern, UK) and the total phenol content was measured by the Folin–Ciocâlteu method [36].

### 2.3. Preparation of CPHE Phenolic Nanopowder/CA Composite Membrane

The CPHE phenolic nanopowder/CA mixed matrix membrane was synthesized by using the dry–wet phase inversion method. Each combination of CA, CPHE nanopowder, and solvents are presented in Table 1. 

Every combination was stirred for 2 h by using a magnetic stirrer until it became homogeneous. The membrane solution was then allowed to stand for 24 h until the air bubbles disappeared. Using a casting knife (Elcometer, Manchester, UK), thickness was set at 300 µm, and the membrane solution was molded and left for 30 s before finally being soaked in distilled water for 10 min, until solidified. The membrane sheet was then immersed in 40 °C distilled water for 1 min to promote membrane relaxation. The solidified membrane sheet was then dried under flown nitrogen gas at a low and constant gas flow rate, until dried.

### 2.4. Membrane Characterizations

The membrane thickness was measured using a micrometer (accuracy of 0.01 mm) by averaging five measurement points at the top, bottom, right, left, and center edges of the membrane sheet. Tensile strength and elongation testing were carried out using a tensile strength instrument (Imada ZP-50N, Toyohashi, Japan). The test was important to measure the mechanical properties of the membranes, which will allow them to be utilized under pressure during the filtration process. The hydrophilicity of the cellulose acetate membranes was measured using a customized contact angle meter. 

Scanning Electron Microscopy (FESEM-FEI Quanta FEG 650, Hillsboro, OR, USA) was used to capture cross-sectional images of membranes and bacterial attachment on the membrane surface. An image cross-section was investigated to link the membrane morphology and its properties, while observation of bacterial attachment on the membrane surface was performed to evaluate the antibiofouling properties of the composite membranes.

### 2.5. Pure Water Flux

**Figure 1:**
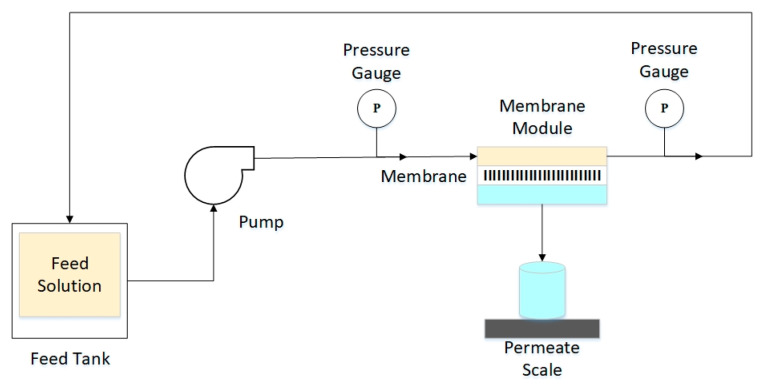
Crossflow membrane filtration system.

The pure water flux of each membrane, synthesized based on the combination presented in Table 1, were investigated by using a custom-made set-up, as shown in Figure 1. The custom-made PMMA membrane cell was used to measure the membrane flux and applied crossflow filtration, similar to previous work, with some modification [13]. A circle membrane coupon with a diameter of 60 mm was placed between the two net-type spacer layers to ensure good fluid motion in the feed and permeate sides. The pure water flux study was performed to assess the transport properties across the membranes. The feed was demineralized water, and pressurized at 0.5 bar by using a pump. The mass of water passing through the membrane or the permeated was measured every 3 min for 30 min. 

Pure water flux was calculated using the following equation:

(1) $$J_{CW}=\frac{Q}{A\cdot \Delta t}$$

where *J_CW_* is the pure water flux (L m^−2^ h^−1^), *Q* is the permeate volume (L), *A* is the effective membrane area (m^2^), and Δ*t* is the running time (h) [37]. 

### 2.6. Antibiofouling Study

The antibiofouling property of the membranes was tested by immersing the membrane sheet into an *Escherichia coli* solution, as conducted by previous studies, with some modifications [38,39]. *Escherichia coli* was selected as a model since the bacteria is widely employed as a water biological contaminant indicator. Bacteria were grown on Oxoid nutrient broth (Thermo Scientific, Waltham, MA, USA) media at 37 °C for 24 h. The prepared bacteria were diluted with buffered peptone water until a suspension of no more than 300 CFU/mL was obtained. Each membrane was cut into 0.5 cm × 0.5 cm sections and soaked in bacterial suspension for 8 h and then taken and preserved using formaldehyde. The fixed attached bacterial cells on the membrane surface were observed and measured by using SEM (FESEM-FEI Quanta FEG 650, Hillsboro, OR, USA).

## 3. Results and Discussion

### 3.1. CPHE Extract Characteristics

**Figure 2:**
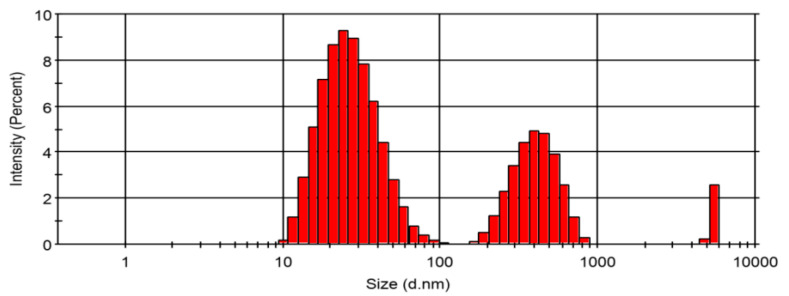
Particle size and distribution of the CPHE phenolic nanopowder.

Following the extraction procedures, CPHE phenolic nanopowder was produced and we measured its particle size and distribution. The results are presented in Figure 2. As shown in Figure 2, CPHE was dominated by particles with an average size of 28.84 nm with an intensity of 67.5%. The remaining 29.7% had an average size of 417.1 nm and 2.8% had an average size of 5501 nm. It is concluded from the measurement that CPHE particles are in the range of a nanoparticle’s size. The polydispersity index (PDI) of the particle size distribution is 0.345. 

Total phenolic content (TPC) of the CPHE nanopowder was measured and have previously been reported [32]. The TPC of CPHE nanopowder is 453 mg GAE/g dw extract, and the in vitro study of antibacterial activity of the CPHE phenolic nanopowder showed a zone of inhibition against *Eschericia coli* at a concentration of 5 mg/mL. The CPHE might also contain terpenoids, alkaloids, and saponins [31].

### 3.2. Composite Membrane Properties

**Figure 3:**
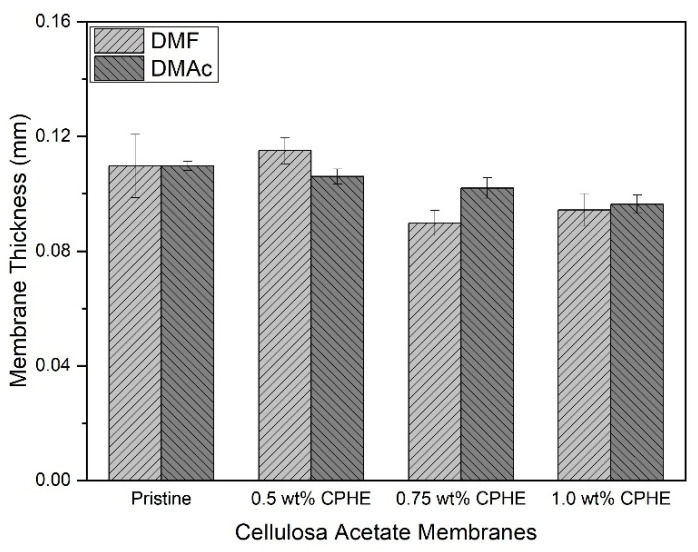
Average membrane thickness with different membrane variations.

The CPHE phenolic nanopowder-impregnated CA composite membrane sheets were prepared using DMF and DMAc solvents. Although the CPHE nanopowder was added, the thickness of the membrane sheet was not changed significantly (Figure 3). As shown in Figure 3, the thickness of the CPHE-impregnated CA composite membranes were slightly reduced compared to the pristine one (both prepared using DMF and DMAc), but almost negligible. The average thickness of the membrane sheets was 0.09–0.11 mm, and comparable with the pristine ones. A similar thickness is important because the mechanical properties and mass-transport properties of the composite membranes were investigated from comparable morphological thickness of the membranes.

**Figure 4:**
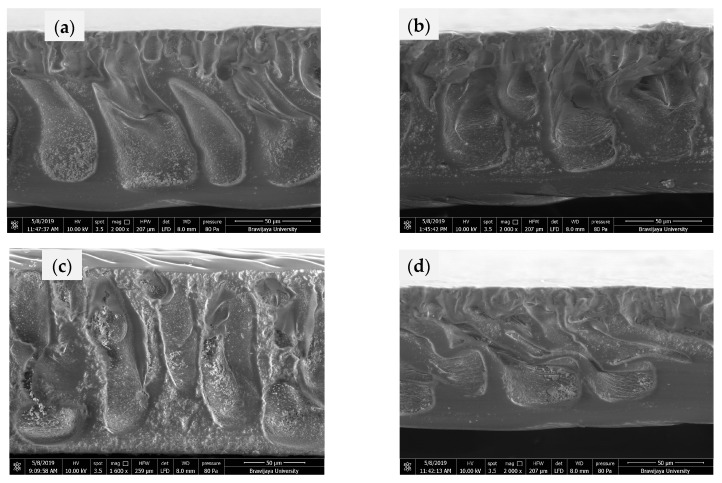
Cross-sectional membrane sheets observed by SEM (2000 × magnification): (**a**) Pristine CA membrane (DMAc); (**b**) 1.0 wt% CPHE/CA composite membrane (DMAc); (**c**) Pristine CA membrane (DMF); and (**d**) 1.0 wt% CPHE/CA composite membrane (DMF).

In order to identify the internal morphology of the cellulose acetate membranes, cross-sectional structure of the membrane sheets were observed using SEM, and shown in Figure 4. As shown in Figure 4, all membranes have a macrovoid structure, with a thinner layer on top of the membrane surface. The addition of CPHE phenolic nanopowder did not affect the structure significantly; however, the CPHE phenolic nanopowder appeared on the surface of the membrane pores. The CPHE nanopowder might prevent the adherence of bacterial cells during biofilm formation on the membrane surface. Although the thickness of the CPHE nanopowder-impregnated CA composite membranes were slightly lower than that of the pristine CA membrane, the difference was not significant. The reduced thickness might be affected by the opening of the polymer matrix due to the presence of the CPHE nanopowder, and promoting a higher releasing rate of the solvent during membrane solidification. 

**Figure 5:**
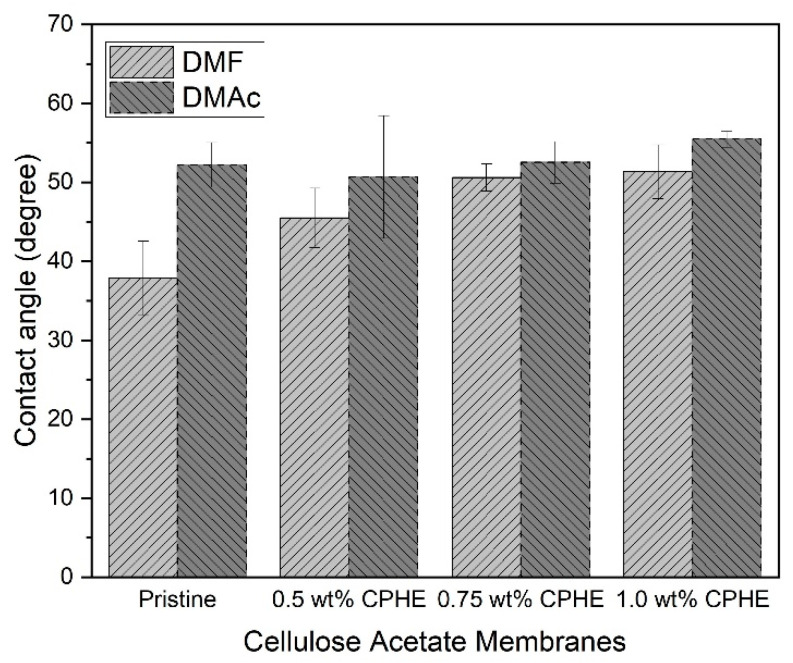
Hydrophilicity of the cellulose acetate membrane sheets.

Moreover, the upper pore layer of the composite membrane surface was more open than that of the pristine membrane [40]. Due to the more open upper pore layer, the permeability of the composite membrane is expected to be higher than that of the pristine membrane. The membrane surface properties are also important to be measured, especially the hydrophilicity. The hydrophilicity is measured by the contact angle measurement, as shown in Figure 5.

As shown in Figure 5, all membrane sheets have a contact angle between 40° and 60°, which means all the membranes are hydrophilic, regardless of the solvents used and the addition of CPHE extracts. DMF and DMAc are polar aprotic solvents, and therefore have hydrophilic properties. However, the DMF has higher polarity compared to the DMAc, possibly affecting the hydrophilicity of the membranes due to hydrogen bonding with water molecules [41,42]. Phenol can interact with water molecules to form a hydrogen-bonded interaction [43]. The effect of a higher phenol concentration may not affect the membrane hydrophilicity, but the membrane surface roughness due to the presence of phenolic powder may affect the water contact angle on the membrane surface [44]. The measurement of membrane hydrophilicity is important, to show that adhesion of bacteria and its eradication is mainly the effect of the CPHE phenolic nanopowder and not caused by the nature of the membrane surface properties, i.e., hydrophilicity. The contact angle measurement also shows that the addition of the CPHE phenolic nanopowder barely affects the membrane surface properties, yet the nano powder was found embedded in the polymer matrix.

**Figure 6:**
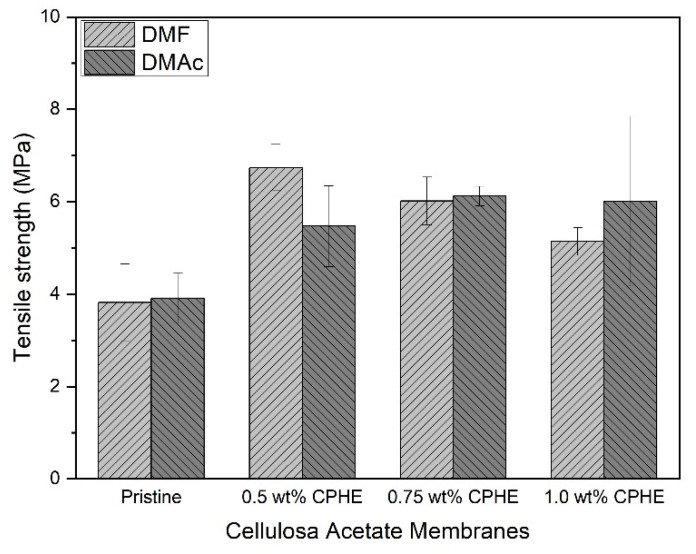
Tensile strength of the cellulose acetate membrane sheets.

In order to check whether the morphology affect the mechanical properties of the membranes, measurements of the membrane tensile strength and elasticity were performed. Figure 6 shows the membrane tensile strength.

As shown in Figure 6, membrane tensile strength was increased when the CPHE phenolic nanopowder was added. The increment tensile strength values of the composite membranes occured both for membranes prepared by using DMF and DMAc. Both with DMF or DMAc solvents, tensile strength values appeared to be better in the presence of CPHE. In addition to causing the macrovoid to shrink, the CPHE nanopowder seemed able to increase the mobility of the CA chain, thereby increasing the mechanical strength in the membrane. According to Zafar et al. [45], the mechanical strength of the CA membrane increases with increasing concentrations of the additives (PEG, PG, and EG) added. However, when the nanopowder was not distributed evenly or formed aggregates, a reduction in the mechanical strength would occur [18].

For membranes prepared with DMF solvents, the tensile strength values increased with the addition of 0.5 wt% CPHE nanopowder. However, the value then decreased when the higher percentage of CPHE nanopowder was added. As for membranes prepared with DMAc, the membrane tensile strength values increased when 0.5 wt% CPHE nanopowder was added, and continued to increase until the addition of 0.75 wt% CPHE nanopowder. However, the more CPHE nanopowder added, the membrane tensile strength decreased (1.0 wt% CPHE nano powder addition). This is due to more nanoparticles that penetrated the CA polymer matrix, and so the polymer chain became disrupted, causing easier breakage [18].

**Figure 7:**
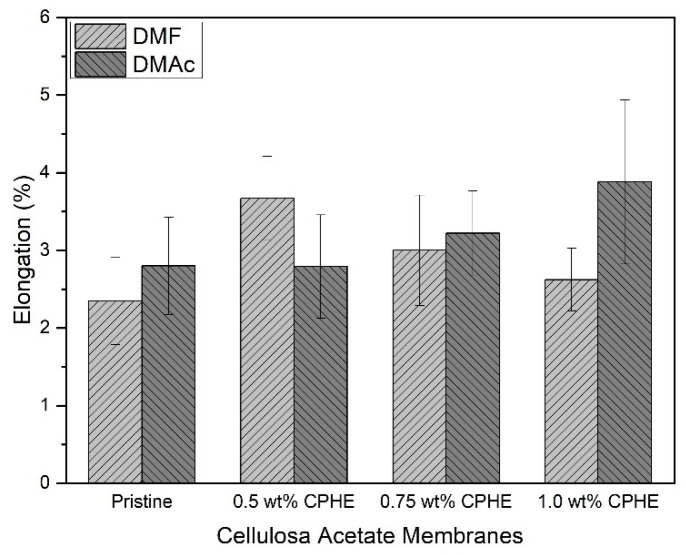
Elongation of the membrane sheets.

Different trends for the membrane elongation value are shown in Figure 7. The membrane elongation was not relatively changed with the addition of the CPHE nanopowder. The values were observed for composite membranes prepared both with DMF and DMAc solvents.

### 3.3. Membrane Volumetric Transport Assessment

Membrane transport is a major property for a polymer membrane sheet since the membrane is used to separate two phases. A membrane must possess a high mass transport for a substance (liquid phase) and prevent another substance (liquid or solid phase) being transported to a different side of the membrane sheet. Pure water flux is used to assess liquid transport over the membrane, and to investigate how the transport occurred. 

The permeability of the membrane sheet is influenced not only by the internal properties of the membrane, such as interactions of pore size (and pore distribution), tortuosity (level of complexity of the porous arrangement), and thickness of the active part of the membrane, but also the external condition applied to the membrane, such as the trans membrane pressure difference between two sides of the membrane. Besides, it will also be greatly affected by fouling and concentration polarization on the membrane active surface [46].

**Figure 8:**
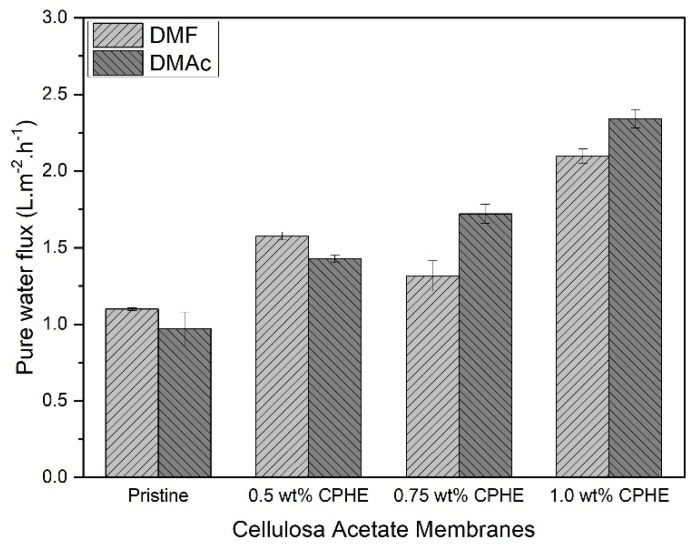
Pure water flux measured on each membrane.

The pure water flux of the CPHE nanopowder-impregnated CA composite membranes and the pristine CA membranes are shown in Figure 8. As expected from the membrane morphology earlier shown in Figure 4, the pure water flux of the CPHE/CA composite membranes was increased (both prepared with DMF and DMAc), and an increment in flux observed when the CPHE nanopowder concentration was increased. The CPHE nanopowder contained phenolic compounds (hydroxyl groups) that are expected to bond with the solvents (DMAc and DMF) through hydrogen bonding, as in the formation of coordinate molecules between the alcohol additives and DMAc solvents on the polysulfone membrane [47]. The existence of these bonds allowed the formation of polymer aggregation in the casting solution and promotes the formation of a larger void, resulting in a higher flux value.

Comparing the solvents, in this study, the pure water flux of the pristine membrane prepared using DMF was higher than that of DMAc. When considering the solubility parameter, the CA polymer is basically closer to the DMAc solvent than that of the DMF solvent. Proximity to the value of solubility is usually able to produce a more porous membrane that promote a higher membrane flux. According to Alvi et al. [48], the permeability of polyethersulfone prepared with DMF solvents is higher compared to NMP (N-methyl-2-pyrrolidone). 

Besides solubility, the behavior of phase separation in forming membrane morphology is also influenced by intrinsic viscosity, bias index, and density [49]. According to Idris et al. [50], although the total solubility parameters of polycarbonate polymers (PC) are closer to DCM (dichloromethane), their permeability values are lower compared to using NMP solvents.

### 3.4. Antibiofouling Property

**Figure 9:**
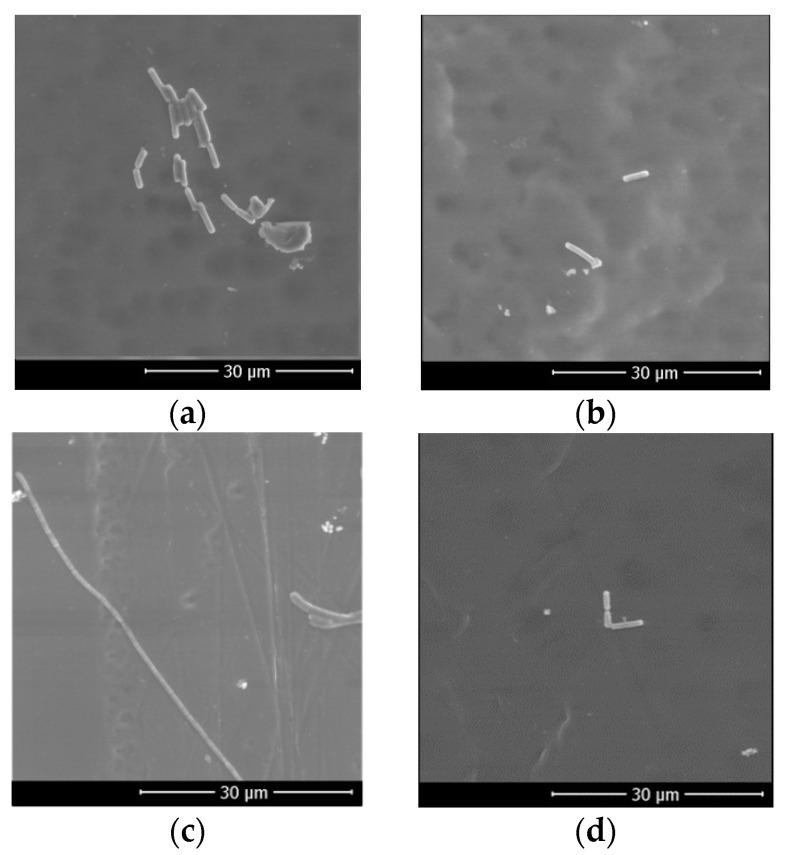
*Escherichiacoli* attachment to the membrane surface: (**a**) Pristine CA membrane (DMAc); (**b**) 1.0 wt% CPHE/CA composite membrane (DMAc), (**c**) Pristine CA membrane (DMF) (**d**) 1.0 wt% CPHE/CA composite membrane (DMF).

The antibiofouling property of the CPHE/CA composite membrane was evaluated by immersing a membrane sheet into an *Escherichia coli* solution for 8 h. For 8 h, it is expected that the bacteria cells will grow and expand their colonies. After fixation by using formaldehyde, the membranes were observed using SEM and the results are shown in Figure 9.

As shown in Figure 9, *Escherichia coli* cells attached to the membrane surface after immersion for 8 h. Within the time span, biofouling predominantly occurred by bacterial cell adhesion. There was no biofilm observed in this very short time span [51]. Both composite membranes were prepared with DMAc and DMF solvents; the number of adhered bacteria cells on the 1.0 wt% CPHE/CA composite membrane surface was less than that attached to the pristine CA membranes. 

**Figure 10:**
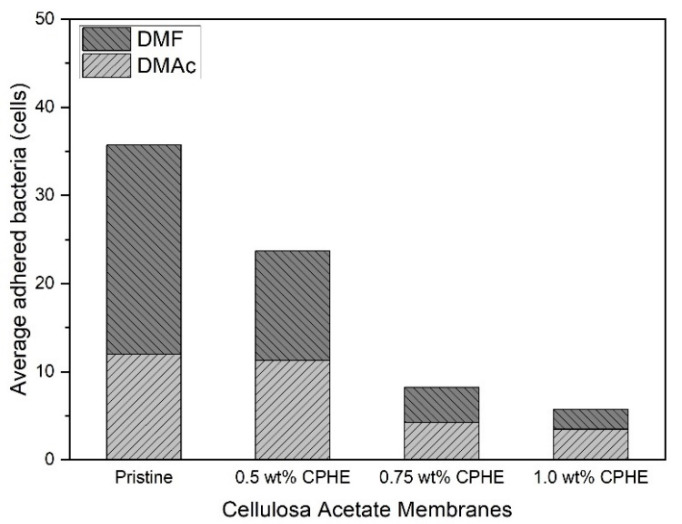
Number of bacteria attached to the membrane.

The measured number of bacteria is presented in Figure 10. As shown by increasing the concentration of the CPHE nanopowder in the CA polymer, the number of bacteria attached was decreasing, regardless of the membranes being prepared by DMF or DMAc. In membranes prepared with DMAc, the addition of 1.0 wt% CPHE nanopowder could reduce the number of adhered bacteria up to 70.8%. While, on a membrane surface prepared with DMF, the addition of CPHE at the same concentration could reduce the number of attached bacteria up to 90.5%.

As an antibacterial agent, phenolic compounds induced protein denaturation, which could promote the cessation of *Escherichia coli* cells’ metabolic activity. Termination of *Escherichia coli* metabolic activity can inhibit their growth, resulting in the death of bacterial cells [52]. At low concentrations, CPHE phenolic compounds activate the most vital enzyme systems in *Escherichia coli* cells. Furthermore, in high concentrations, CPHE phenolic compounds are able to disrupt and penetrate cell walls, which then denaturate proteins in bacterial cells [53].

The phenolic compounds in CPHE nanopowder are natural antibacterial compounds. In general, natural antibacterial activities have a limited time span. Therefore, to find out the effective time span of the CPHE antibacterial activity on a CA membrane, further studies need to be done; for instance, with a longer time span, and by performing the actual membrane application (in food processing). A combination with physical cleaning by using gas sparging can potentially be used, as the CPHE could destroy the bacterial cells and the gas/liquid shear could remove the bacterial debris without any biofilm formed on the membrane surface [54,55,56].

In addition, in this study, it is unknown whether the bacteria that attached to the CPHE/CA composite membranes actually died or only were inhibited during their replication. Therefore, it is necessary to do further tests using other methods to find out the exact mechanism of the CPHE nanopowder in controling the amount of bacterial attachment to the membrane; for instance, with total plate count (Xia et al. [38]) and confocal laser scanning microscopy [57].

## 4. Conclusions

A CPHE/CA composite membrane was successfully synthesized via a phase inversion process. Different solvents, in this case, DMF and DMAc, were used. The results showed that the addition of CPHE phenolic nanopowder was able to improve the mechanical characteristics of the cellulose acetate membrane both using DMF and DMAc solvents. The highest pure water flux value was 2.34 L m^−2^ h^−1^, achieved by 1.0 wt% the CPHE/CA composite membrane prepared with DMAc. The CPHE nanopowder was also able to reduce the amount of *Escherichia coli* attached to the CA membrane by 70.8% (DMAc) and 90.5% (DMF). The results show that the CPHE phenolic nanopowder can be used to control biofouling in membrane applications. However, further study is required to investigate the underlying mechanism of the CPHE phenolic nanopowder in controlling biofouling.

## Figures and Tables

**Table 1:** Combinations of CPHE nanopowder, CA, and solvents (dimethylformamide (DMF) or dimethylacetamide (DMAc)) used in this study.

Membrane Formation	Solvent	CA (g)	CPHE (g)	DMF (mL)	DMAc (mL)
Pristine CA	DMF	4	-	20	
CA + 0.5 wt% CPHE	DMF	3.98	0.02	20	
CA + 0.75 wt% CPHE	DMF	3.97	0.03	20	
CA + 1.0 wt% CPHE	DMF	3.96	0.04	20	
Pristine CA	DMAc	4	-		20
CA + 0.5 wt% CPHE	DMAc	3.98	0.02		20
CA + 0.75 wt% CPHE	DMAc	3.97	0.03		20
CA + 1.0 wt% CPHE	DMAc	3.96	0.04		20
